# Thermo-Optoplasmonic Single-Molecule Sensing on Optical Microcavities

**DOI:** 10.1021/acsnano.4c00877

**Published:** 2024-06-26

**Authors:** Nikita A. Toropov, Matthew C. Houghton, Deshui Yu, Frank Vollmer

**Affiliations:** † Department of Physics and Astronomy, University of Exeter, Exeter EX4 4QD, U.K.; ‡ Optoelectronics Research Centre, University of Southampton, Southampton SO17 1BJ, U.K.; § Qingdao Innovation and Development Center, Harbin Engineering University, Qingdao, Shandong 266000, China; ∥ Department of Life Sciences, University of Bath, Bath BA2 7AX, U.K.; ⊥ National Time Service Center, Chinese Academy of Sciences, Xi’an 710600, China

**Keywords:** microresonator, plasmon, protein, tryptophan, sensor, absorption

## Abstract

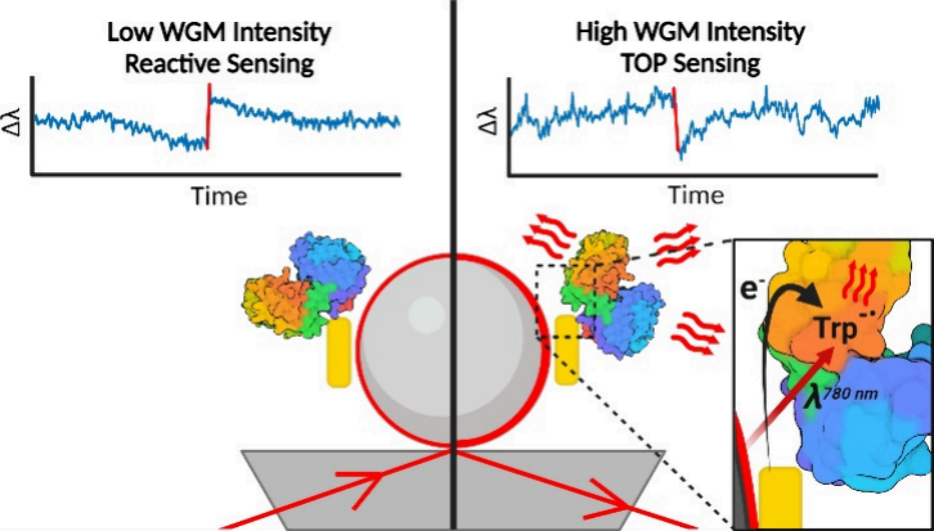

Whispering-gallery-mode (WGM) resonators are powerful instruments for single-molecule sensing in biological and biochemical investigations. WGM sensors leveraged by plasmonic nanostructures, known as optoplasmonic sensors, provide sensitivity down to single atomic ions. In this article, we describe that the response of optoplasmonic sensors upon the attachment of single protein molecules strongly depends on the intensity of WGM. At low intensity, protein binding causes red shifts of WGM resonance wavelengths, known as the reactive sensing mechanism. By contrast, blue shifts are obtained at high intensities, which we explain as thermo-optoplasmonic (TOP) sensing, where molecules transform absorbed WGM radiation into heat. To support our conclusions, we experimentally investigated seven molecules and complexes; we observed blue shifts for dye molecules, amino acids, and anomalous absorption of enzymes in the near-infrared spectral region. As an example of an application, we propose a physical model of TOP sensing that can be used for the development of single-molecule absorption spectrometers.

## Introduction

Photonic sensing of single molecules is becoming a well-established scientific direction, providing powerful instruments for biological and medical sciences. Optical techniques have made it possible to experimentally observe and manipulate single nanoparticles, molecules, and atomic ions.^1−7^ Examples of different physical mechanisms for single-molecule studies include single-molecule imaging by optical absorption,^5,6^ photothermal detection scheme,^8^ plasmon-based photothermal spectroscopy,^9^ light-scattering-based techniques,^10^ manipulations with optical tweezers,^1^ fluorescent microscopy,^11^ and several others. A noticeable contribution to the optical detection of single molecules was brought by whispering gallery-mode (WGM) resonators. Such resonators were initially described by Lord Rayleigh in 1910;^12^ to date, optical microresonators are known as having unsurpassed quality (*Q*) factors, up to 10^10^,^13^ making them very sensitive to small environmental perturbations. Their basic sensing principles (resonant mode change evaluation) exploit the seminal theory proposed in the 1940s by Bethe and Schwinger.^14^

One of the most common shapes of optical microresonators used for biosensing is spheres, since they are relatively easy to fabricate from standard optical fibers and possess high *Q*-factors.^4^ However, their effective mode volumes are relatively large: for high-*Q* spherical resonators of up to 100 μm in diameter, the mode volume at near-infrared probing wavelengths reaches ∼10^3^ μm^3^, which was a limiting factor for detecting molecules below the size of a monolayer.^15,16^ To achieve better localization of probing light, it was proposed to decorate WGM resonators with metal nanoparticles of ∼10 nm, supporting localized plasmonic oscillations.^17^ WGM resonators coupled to plasmonic nanoparticles, known as optoplasmonic sensors, form the basis of new applications of WGM in sensing. Optoplasmonic single-molecule sensing becomes feasible due to the proportional perturbation of the optical microcavity induced by polarizable molecules like proteins, in tandem with the near-field enhancement of the plasmonic nanoparticle such as a plasmonic nanorod.^2,3^ Recent examples of optoplasmonic sensor applications have demonstrated the detection of molecular movements in solutions diluted to attomolar concentrations^18^ and studying single-molecule thermodynamics and conformational changes of proteins.^19,20^ There are bright prospects for single-molecule studies with optoplasmonic WGM, from advancing single-molecule investigations to specific spectral fingerprinting of molecules.^21^ Notably, all-dielectric WGM microtoroidal resonators have already been used for single-particle photothermal absorption spectroscopy of nanoparticles^22^ and single molecules.^23^ Despite this, the findings regarding single molecules, as reported by Armani et al. in their publication, were subsequently subjected to additional scrutiny through theoretical calculations in refs (24 and 25).

We propose single-molecule detection on the optoplasmonic WGM sensor using the thermo-optical effect initiated by single molecules binding to a plasmonic nanorod. This method represents a departure from the prevailing trend of utilizing low-intensity light for sensing of single quantum objects, down to single photons.^26−28^ Instead, our approach demonstrates optical single-molecule sensing at comparatively higher power levels, leading to the discovery of the thermo-optoplasmonic (TOP) biosensing mechanism. Indeed, optoplasmonic sensing experiments have mostly been performed at low intensities of WGM excitations (1–100 μW). Nevertheless, it is important to highlight that optoplasmonic sensors typically exhibit a linear response to molecules binding to plasmonic nanoparticles. This indicates that the sizes, numbers, and optical properties of the objects being studied lead to proportional red (or blue) WGM resonance wavelength shifts based on their polarizability. Specifically, molecules such as DNA and protein with an excess polarizability in water induce a red shift in the resonance wavelength, known as the reactive sensing mechanism.^29,30^ Herein, we reveal that increased intensity of WGM leads to disproportional and sign-changed resonance wavelength shifts in optoplasmonic single molecule detection, which subsequently can be used to estimate the absorption cross-section of single molecules. For this, we built an optoplasmonic sensor with WGMs excited at near-infrared wavelength and gold nanoparticles with near-infrared plasmon resonances to study single-protein attachment events. Changing the parameters of light coupling to the WGM resonator, the *Q* factor of the sensor, and the exciting intensities, we achieve a high intensity of light that activates thermal hotspots when single proteins attach to the plasmonic nanoparticle, providing information about their absorption. The universality of this technique is confirmed directly via studying binding events for seven types of molecules and complexes: unadulterated proteins, Alexa Fluor 790 (Alexa) conjugated proteins, pure solution-based Alexa molecules, amino acid molecules, and solution-based IRdye 800CW (IRDye) molecules (Table 1).

**Table 1:** Characteristic Values of the Tested Molecules and Complexes

**Sample**	**3PGK**	**3PGK–Alexa**	**Adk**	**Adk–Alexa**	**Tryptamine**	**Alexa**	**IRDye**
**Absorption peak, nm**	276	779	271	777	296	780–784	778
**Molecular weight, Da**^a^	44,376	46,126	23,999	25,749	160	1,750	1,166
**Extinction coefficient at 280 nm,cm^–1^ M^–1^**	22,920	43,700	10,430	31,200	5,405	20,784	7,200
**Extinction coefficient at 780 nm,cm^–1^ M^–1^**	∼0	167,000	∼0	95,000	∼0	260,000	240,000
**No. of tryptophan residues (indole rings)**	2 (2)	2 (2)	0	0	(1)	b	–

a As predicted by the ExPASy ProtParam online tool.^31^

b Alexa Fluor 790 structure unpublished.

## Results

### Optoplasmonic Sensing of Proteins

Four protein samples were investigated at the first stage: 3-phosphoglycerate kinase (3PGK), adenylate kinase (Adk), and 3PGK and Adk conjugated with Alexa, respectively; protein labeling and other experimental protocols are provided in the Methods.

**Figure 1:**
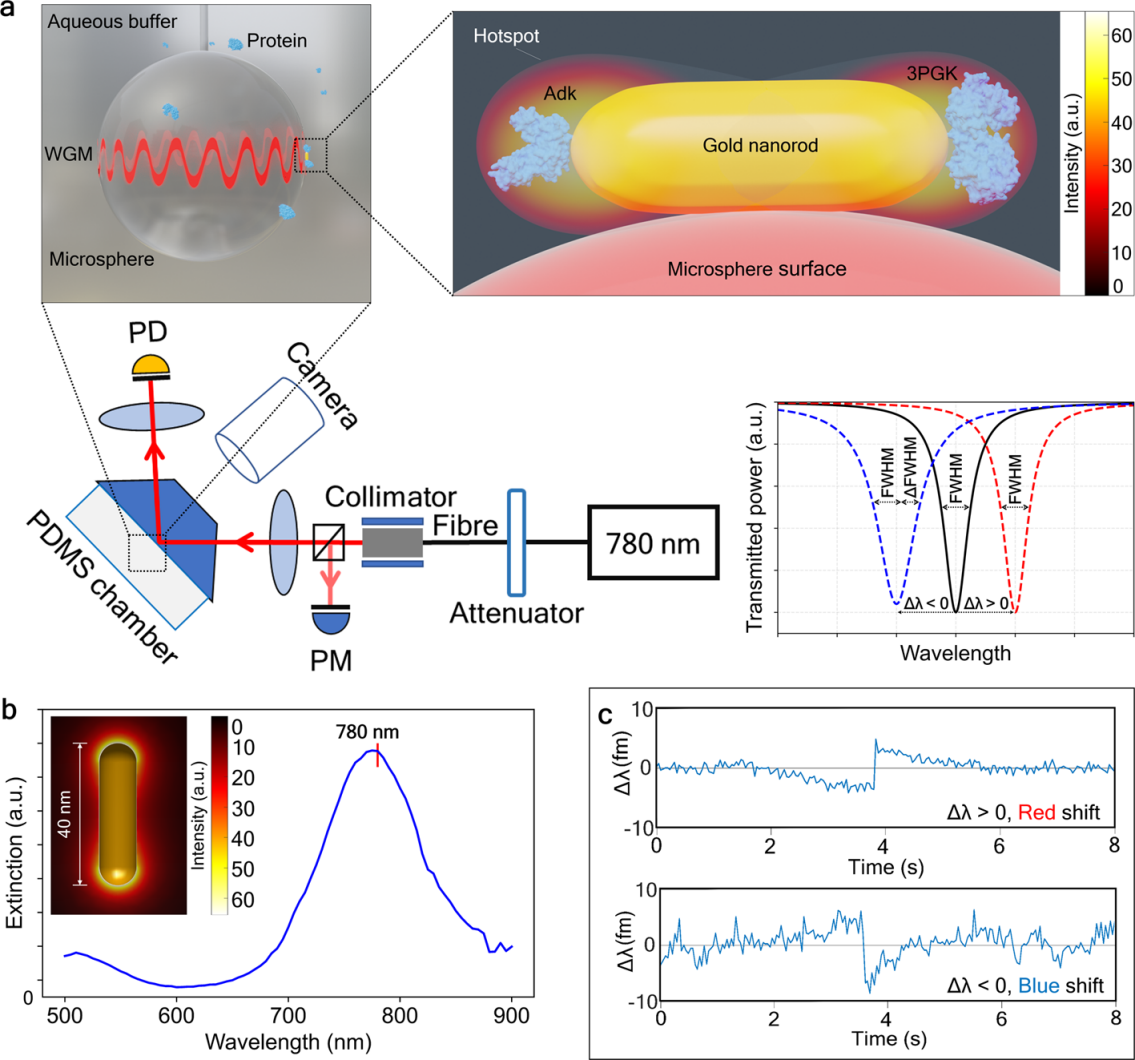
Optoplasmonic sensing. (a) Optoplasmonic single-molecule sensor scheme. A collimated 780 nm laser beam passes through a 90/10 beam splitter: 90% - to a 50 mm focusing lens, 10% - to a power meter (PM). The incident beam (≈6°) reflects from the back side of a prism, inducing evanescent waves that excite whispering gallery modes in a ∼90 μm-diameter silica microsphere placed behind the prism. The reflected beam is focused on a detector (PD); WGMs are observed as dips in the transmission spectrum of the system. A PDMS chamber containing analyte molecules is attached to the back side of the prism. Resonance wavelength shift (Δλ) and full width at half-maximum (Δfwhm) of WGMs are tracked with the photodetector, connected to a data acquisition card (DAQ). Gold nanorods are attached to the microsphere surface. Protein samples (Adk or 3PGK; conjugated or not with Alexa Fluor 790) bind to these nanorods at the tips within an enhanced electric field that can detect perturbations of polarizability and hence the presence of protein molecules. (b) Extinction spectrum of gold nanorods used in experiments. Inset: electric field distribution around the nanorod with the LSPR at 780 nm. (c) Examples of measured resonance wavelength traces showing red, Δλ > 0, and blue, Δλ < 0, wavelength shifts under the attachment of 3PGK molecules at low and high intensities of WGM, respectively.

In our experiments, we recorded the attachment events for each molecule type within the single-molecule regime of optoplasmonic sensors. The confirmation of the single-molecule regime was established by examining survival plots, as detailed in the Supporting Information and similar to the approach described in ref (3). The principal experimental scheme is illustrated in Figure 1a. Spherical WGM resonators were made by melting SMF-28 optical fibers with a CO_2_-laser. To achieve high *Q*-factors of WGMs, radii of resonators were set to 45 ± 8 μm. WGMs were excited with a 780 nm emission of a cw tunable diode laser via a prism coupler at a wide range of power levels (0.01–5.5 mW) and coupling efficiencies (6–45%). The chamber, containing the WGM resonator and of ca. 300 μL volume, is formed of a polydimethylsiloxane (PDMS) polymer sandwiched between the prism and a microscope cover glass slide. The laser was connected to a fast-speed data acquisition card (DAQ), which synchronizes the laser wavelength scanning (50 Hz) and a photodiode with a PC via a LabVIEW program when recording the transmission spectrum. WGM frequency shifts were tracked in a spectral range of several picometers around a resonance line, while the resonance full width at half-maximum (fwhm) changes were also recorded. The system allows one to achieve ∼1 fm in spectral resolution and 20 ms in time resolution.

We used an established, one step wet chemical procedure for attaching gold nanorods to the silica microsphere to assemble the optoplasmonic sensor, based on Baaske et al.^2^ The attachments of ∼5 plasmonic gold nanoparticles, with a longitudinal LSPR (localized surface plasmon resonance) peak at 780 nm (Figure 1b), were detected from step signals in the sensor response, mediated by a low-pH HCl solution, as a first step of the experiment. The size of the nanorods was 10 nm × 38 nm, providing a negligible effect on WGM propagation; therefore, we did not observe reflected modes or mode splitting.^32^ Nanorod binding increases fwhm (full width at half-maximum) values by roughly 100 fm (i.e., slightly reducing *Q*-factors) and depending on the nanorod orientation upon attachment (Supporting Information^2^), though it allows WGM sensors to be capable of detecting single molecules. In the next step of the experiment, analyte molecules (Table 1) chemically react with the attached nanorod by thiol reaction with gold, except for 3PGK which utilizes a nickel-NTA linker (see the Supporting Information).

### 3PGK and 3PGK–Alexa

**Figure 2:**
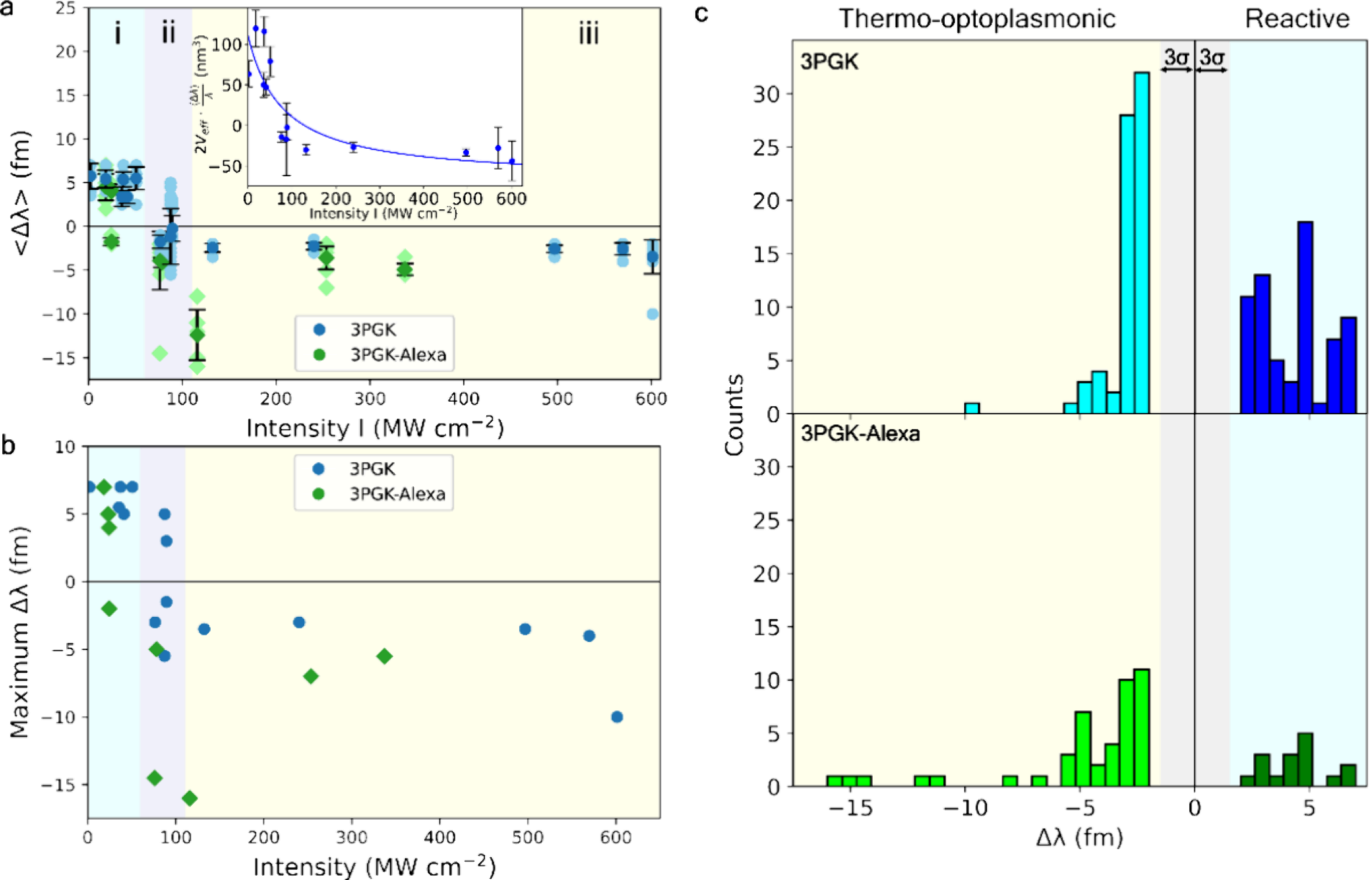
Single-molecule detection of 3PGK and 3PGK–Alexa. (a) Optoplasmonic sensing of 3PGK (mean, dark blue circles; raw data, light blue circles) and 3PGK–Alexa (mean, dark green diamonds; raw data, light green diamonds). Averaged values ⟨Δλ⟩ of WGM wavelength shifts depend on the evanescent intensity *I* of the WGM around Au nanorods. Regions (i), (ii), and (iii) represent reactive sensing, near-zero shifts, and blue shifts, respectively. Inset: Dependence of 2*V*_eff_·⟨Δλ⟩/λ with the effective mode volume *V*_eff_ and wavelength λ = 780 nm on *I*. Symbols correspond to the experimental data, while the line gives the curve fitting based on eq 1—*R*^2^ = 0.6946. (b) Maximal wavelength shifts vs the evanescent intensity *I*. Maximal wavelength shifts show binding at the tips of nanorods, when nanorod longitudinal plasmonic modes are effectively excited. (c) Histograms of wavelength shifts Δλ. 3PGK shifts grouped into the reactive sensing mechanism (red shifts, blue) and the thermo-optoplasmonic mechanism (blue shift, cyan). The same for 3PGK–Alexa is shown below: red shifts (green) and blue shifts (lime). The gray area indicates significance levels of triple the standard deviation (3σ).

Single-molecule biosensing is based on tracking WGM resonance changes, Δλ, under attachment events (Figure 1c). In our experiments, 3PGK molecules from *Geobacillus stearothermophilus* were selectively bound to the gold nanorods. Generally, under attachment events, WGM resonances are either red (Δλ > 0) or blue (Δλ < 0) shifted in relation to their initial positions, depending on the values of nanoparticle or molecular polarizability.^33^ However, our experiments revealed that wavelength shifts can be red or blue for the same molecules, where these shifts are mainly governed by changes of local refractive indexes. Figure 2 shows the dependence of the sign of the wavelength shift on the intensity and represents an intensity-dependent diagram of single-molecule sensing with optoplasmonic sensors. For this diagram, the local evanescent intensity *I* at the tips of the nanorods (the location where the binding of single molecules can be detected^2,3^) was calculated by considering the effective mode volumes of TE equatorial modes, power of exciting beams, coupling values, WGM *Q*-factors, and field enhancement around plasmonic nanorods (see the Methods). The figure can be subdivided into three sections: reactive sensing (red shifts), near-zero shifts, and blue shifts.

#### i Reactive Sensing Mechanism

**Figure 3:**
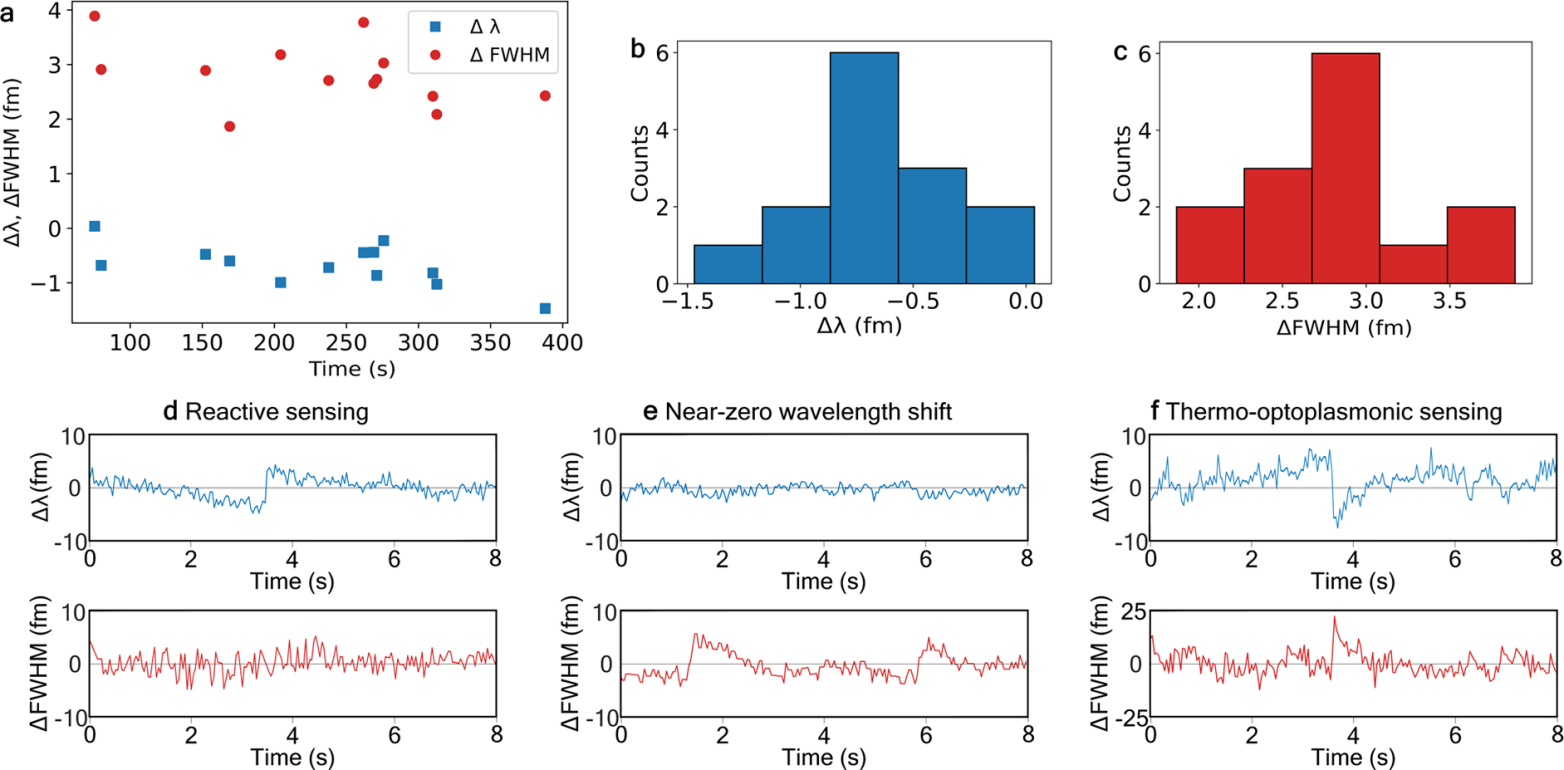
Sensing of single 3PGK molecules. (a) 3PGK optoplasmonic sensing at the evanescent intensity *I* = 89.3 MW cm^–2^. Near-zero resonance wavelength shifts Δλ (blue squares) are accompanied by the changes of full width at half-maximum (Δfwhm) (red circles). Changes of the resonance wavelength Δλ are below the noise level (i.e., triple standard deviation of 3σ) and are considered to be effectively zero. (b) Histogram of the number of attachment events (counts) vs wavelength changes Δλ. Majority of shifts found between −0.8 and −0.6 fm, well below the noise level (3σ = 1.5 fm). (c) Histogram of the number of attachment events (counts) detected via fwhm changes vs Δfwhm. Majority found between 2.75 and 3 fm, greater than the noise level (3σ = 1.8 fm). (d–f) Examples of wavelength shifts and fwhm changes. (d) 3PGK reactive sensing with Δλ > 0 and Δfwhm ≈ 0. (e) Near-zero wavelength shift with Δλ ≈ 0 and Δfwhm > 0. (f) Thermo-optoplasmonic sensing with Δλ < 0 and Δfwhm > 0.

The first section of the diagram (Figure 2a, i, and Figure 2b), ending at *I* ∼ 60 MW cm^–2^, corresponds to the conventional reactive sensing mechanism^2,34^ when binding events cause changes of the resonant wavelength—positive wavelength shifts Δλ > 0, as presented in Figure 1c. During attachment events, 3PGK molecules cause a strong response, changing the polarizability in the evanescent field of the plasmonic nanorod coupled to the WGM resonator. At such intensity levels, 3PGK binding events demonstrate, independent of the evanescent intensity, wavelength shifts Δλ equal to 6 fm, with standard deviations of signals (σ) within 1 fm (Figure 2c). Extracting signals from the WGM transmission spectra measured with optoplasmonic sensors is described in the Methods. Notably, the attachment events of molecules to nanorods at low intensities of WGM do not significantly affect the spectral width (fwhm) of the resonances (Figure 3d). Variation of the step heights of wavelength shifts seen for resonators of the same size are attributed to differences in the nanorod binding location with respect to the WGM field profile and binding orientation of the nanorod with respect to the WGM polarization.^2^

#### ii Near-Zero Wavelength Changes

Region ii of Figure 2a reflects significant changes in the mechanisms of sensing, where the wavelength shifts become near-zero (Figure 3a, b) or protein attachment becomes effectively undetectable by resonance wavelength changes. Near-zero wavelength changes occur at specific WGM intensities where the reactive sensing (positive effect on Δλ) and TOP sensing (negative effect on Δλ) regimes balance, canceling each other out and resulting in a net-zero effect on WGM Δλ. However, the single-molecule attachment events were still observable via step-like changes of fwhm (Figure 3a, e). The fwhm of the resonances becomes wider by 3 fm on average (Figure 3c), relating to increased losses of the WGM resonator. Note that the fwhm at lower intensities was measured as nonresponsive to the binding events of 3PGK molecules (Figure 3d). The difference of fwhm behavior can be elucidated by considering the *Q*-factors of WGM resonators. These take into account several factors, including most notably: scattering, material losses, losses related to resonator radii, and radiation losses.^20^ Attachment of 3PGK to optoplasmonic sensors does not lead to changes in the resonator’s geometry and therefore, at low intensities, does not contribute to resonator scattering, and fwhm will not change during binding events (we presume that molecular scattering under attachment events could change values of fwhm but within 3σ). *Q*-factors and fwhm are not usually related to absorption of WGM energy by the molecules under test.^20^ However, in the present case where fwhm changes occur at high WGM intensities when 3PGK binds (Figure 3f), absorption of WGM energy is the only factor that can be considered to cause the fwhm response. This demonstrates that thermo-optical sensing can therefore be used for direct estimation of absorption by the molecules.

#### iii Blue Shift

Effectively, the second (ii) and third (iii) regions of Figure 2a represent the same mechanism of WGM resonance changes (i.e., absorptive properties of molecules). The third region of the diagram (Figure 2a, iii) represents blue WGM resonance wavelength shifts observed as binding events of 3PGK molecules to Au nanorods at higher local intensity (*I* > 89.3 MW cm^–2^). The dependence of the sign of wavelength shifts on the intensity reveals a mechanism of single-molecule detection with optoplasmonic sensors, which is related to the strong mutual influence of the intensity of WGMs and molecules under test. Binding events of 3PGK molecules to the nanorod appear as blue shifts (Δλ < 0) accompanied by the partial absorption of optical energy by different amino acids of the 3PGK molecule (Δfwhm > 0); see Figure 3f. Under normal conditions, when intensities are relatively low, 3PGK molecules have a strong absorption band centered at 276 nm, which corresponds to the absorption by the aromatic amino acids of 3PGK molecules: tryptophan, phenylalanine, histidine, and tyrosine (see the Supporting Information). Our results reveal that, when a single 3PGK molecule is attached to a single plasmonic nanoparticle, it also demonstrates absorption bands in the near-infrared spectrum. One plausible mechanism for these new bands relies on the tryptophan residues. In far-field spectroscopy, tryptophan molecules have absorption bands in the UV range, around 280 nm. However, tryptophan molecules may demonstrate red-shifted bands under strong perturbation caused by Trp radical formation on a plasmonic nanoparticle (please see details in the Tryptamine section below).^35^

In our model, after a 3PGK binding event, energy is absorbed by the Trp radical in the 3PGK molecule, which relaxes to the ground state by heat release, causing a local temperature increase of the water around the binding location, the time scale of which is unresolved because of the limited time resolution of the sensor (20 ms). Local heating of water makes the local refractive index smaller (water has a negative d*n*/d*T*). Such localized heating of water results in blue resonance wavelength shifts. This reveals the sensing mechanism, which is caused by thermal changes initiated by the binding of molecules on higher-intensity optoplasmonic sensors, or TOP sensing. TOP sensing, despite employing a distinct plasmon-enhanced mechanism and resulting in the reported negative wavelength shifts, shares similarities with the thermo-optic mechanism proposed by Armani et al.^23,24^ Theoretical confirmation of the single-molecule findings in the context of Armani et al.’s work is still ongoing.^24,25^

Including the local thermal effect, the average value ⟨Δλ⟩ of resonance wavelength shifts induced by single protein molecules binding within the plasmon-enhanced near field (aka plasmonic hotspot) of the optoplasmonic sensor is formulated as (see the Methods)
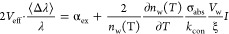
1with the effective mode volume *V*_eff_ of WGM, the excess polarizability α_ex_ and absorption cross-section σ_abs_ of the protein, the refractive index *n*_w_(*T*) of water at temperature *T*, the water’s thermal conductivity *k*_con_ (in units of W m^–1^ K^–1^), the effective volume of the heated water *V*_w_, and the effective heat transferring length ξ. It should be noted that, unlike the conventional definition of the mode volume of WGM,^36^*V*_eff_ here is defined based on the local light intensity at the nanorod’s hotspot (see the Methods) and it has already included the LSPR-induced enhancement of the local electric-field intensity of the gold nanorod. The left-hand side of eq 1 is completely related to the microcavity (e.g., the mode volume and the resonance shift), and all environmental perturbations appear on the right-hand side of eq 1. Two facts contribute to the resonance shift ⟨Δλ⟩ of WGM: (i) As usual, ⟨Δλ⟩ may arise from the excess polarizability α_ex_ of the protein changing the local refractive index of the WGM microcavity. (ii) The bound molecule absorbs the light energy, raising the local temperature and changing the refractive index of the microcavity’s surrounding medium (i.e., aqueous buffer), resulting in an extra resonance shift. Compared to the thermal effect of water, the thermo-optic effect of the microsphere is negligible because of the small rate of change of the refractive index of the microsphere with respect to the temperature (see the Methods). In the low-intensity limit *I* ∼ 0, 2*V*_eff_·⟨Δλ⟩/λ approaches α_ex_ and is positive. As *I* is enhanced, the thermal-effect-induced resonance shift component grows and the positive value of 2*V*_eff_·⟨Δλ⟩/λ is reduced. For a large enough *I*, 2*V*_eff_·⟨Δλ⟩/λ becomes negative. In general, the effective heat transfer length ξ depends on the local electric-field intensity *I*. Under the linear approximation, we express ξ as ξ = ξ_0_ + *βI*, where the constant ξ_0_ approximates the radius of the protein molecule (i.e., the heat transferring distance cannot be smaller than the size of the heat source) and the parameter β may be derived from the curve fitting. Substituting the typical values of *n*_water_ ∼ 1.33,  K^–1^, *k*_con_ = 0.6 W m^–1^ K^–1^ (taken from refs (37−39)), *V*_w_ = 3.3 × 10^–23^ m^3^, and ξ_0_ = 3.5 nm for 3PGK, we obtain α_ex_ = 1.1 × 10^–25^ m^3^ with the 95% confidence interval (0.7 × 10^–25^, 1.6 × 10^–25^) m^3^, σ_abs_ ∼ 7.5 × 10^–16^ cm^2^ with the 95% confidence interval (4.2 × 10^–16^, 10.7 × 10^–16^) cm^2^, and β = 0.045 nm/(MW/cm^2^) with the 95% confidence interval (0.034, 0.056) nm/(MW/cm^2^) from the curve fitting (Figure 2a). The large absorption cross section for 3PGK at 780 nm is observed for the enzyme molecules attached to plasmonic nanorods, providing enhanced near fields of optoplasmonic microcavities at sufficient optical power, suggesting that molecular transitions are excited in the TOP sensing approach that are normally weak in standard absorption spectrometry (see Table 2). Additionally, it is worth noting that the effect of the plasmon resonance shift (resulting from the single-molecule-induced resonance shift) of gold nanorods on single-molecule sensing is negligible (see the Supporting Information).

**Table 2:** Calculated Absorption Cross Sections

**Sample**	**3PGK**	**Tryptamine**	**Alexa790**	**IRDye800**
**σ**_**abs**_**by Spectroscopy at 780 nm, cm**^**2**^	9.69 × 10^–21^	1.12 × 10^–20^	9.93 × 10^–16^	9.17 × 10^–16^
**σ**_**abs**_**by TOP sensing at 780 nm, cm**^**2**^	7.5 × 10^–16^	3.9 × 10^–16^	2.0 × 10^–15^	3.1 × 10^–15^

To confirm the mechanism explained, a series of experiments were performed for 3PGK complexes, where a dye (Alexa Fluor 790) with a molecular absorption peak spectrally close to the maximum of the WGM wavelength, i.e., 780 nm, was selected to be covalently attached to 3PGK. The result of single-molecule 3PGK–Alexa binding is plotted in Figure 2a and b as green dots. The results obtained were similar to experiments with unlabeled 3PGK, where positive wavelength shifts at lower intensities were obtained. They have the same values, 6 fm, when sensing by the reactive mechanism at low *I* because the structure of 3PGK and molecular weight were changed insubstantially by the Alexa label (Table 1). The WGM wavelength changes are switched to blue shifts at higher intensities of WGMs but with greater magnitude than unlabeled 3PGK. In fact, 3PGK–Alexa molecules show blue shifts at even lower intensity than nonlabeled 3PGK molecules. The values of resonance shifts demonstrate big variation, likely related to the different positions of molecules on the nanorod; Figure 2b shows maximal wavelength shifts corresponding to the position of 3PGK at the tips. Under these conditions, the previously nonobservable optical transitions in tryptophan have quite similar values of wavelength shifts to their counterparts in Alexa Fluor 790. The greater magnitude of negative shift and TOP sensing at lower *I* is due to increasing the absorption of the 3PGK molecules by conjugating Alexa, creating an additive effect to enhance the TOP mechanism.

We exclude from our aforementioned analysis the possible fluctuations of temperature due to nanorod heating effects because the proteins are added to the chamber and detected when the sensor is in the steady-state temperature regime. Nonetheless, the increased intensity of WGM causes increased background heating of nanorods (see the Supporting Information), that could make blue shift values upon protein binding smaller at higher intensities. We also consider that increased temperature due to WGM radiation absorption may also cause an increase in temperature of the microsphere. However, using values of thermal conductivities of water, *k*_w_ = 0.6 W m^–1^ k^–1^, and silica, *k*_s_ = 1.38 W m^–1^ k^–1^, supposing that the local temperature of silica is the same as the local temperature of water (i.e., the local temperature increase in silica is the same as the local temperature increase in water) under the thermal equilibrium, we came to the conclusion that the change of the refractive index of water with respect to the temperature is  K^–1^. The change of the refractive index of silica with respect to the temperature is  K^–1^.  is 1 order of magnitude larger than . That is to say, under the same local temperature increase, the change of the refractive index of water is 10 times larger than that of silica. According to this, the effect of the silica temperature increase on the resonance shift, which is generally a competitive process, is much smaller in comparison to the water temperature increase.

### Adk and Adk–Alexa

**Figure 4:**
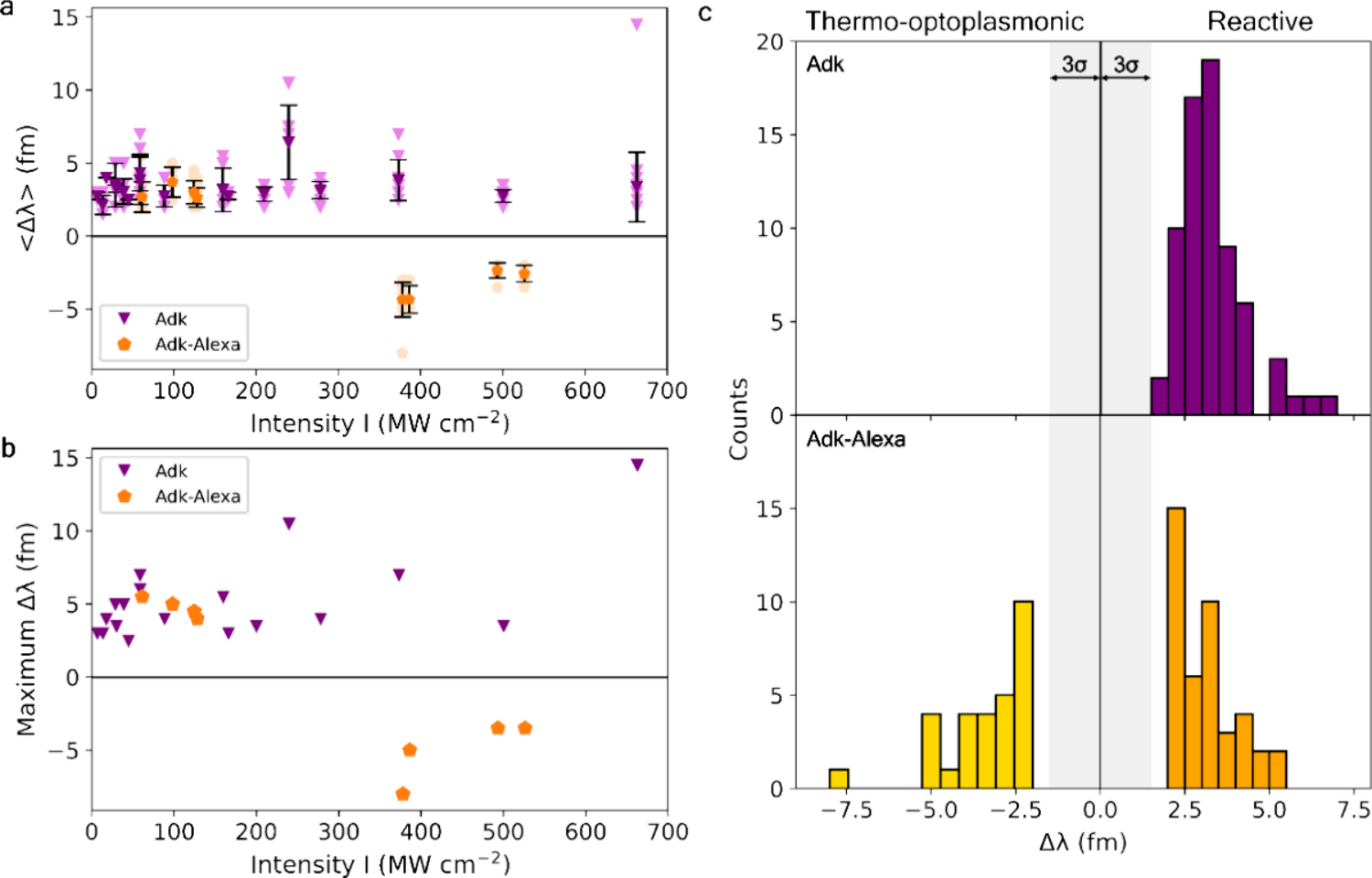
Single-molecule detection of Adk and Adk–Alexa. (a) Optoplasmonic sensing of Adk (purple triangles) and Adk–Alexa (orange pentagons). Averaged values of WGM wavelength shifts ⟨Δλ⟩ depend on the evanescent intensity *I* of the WGM around Au nanorods. (b) Maximal wavelength shifts at each intensity *I* of WGM. Maximal wavelength shifts show binding at the tips of nanorods. (c) Histograms of wavelength shifts. Histograms of Adk (upper) binding shifts by the reactive mechanism (purple). Adk–Alexa (lower) groups are reactive sensing (orange) and blue shifts (gold). The gray area indicates the noise levels of triple standard deviation, 3σ.

To provide more information about TOP sensing, we investigated another protein (*Aquifex aeolicus* Adk) and a protein–dye complex (Adk–Alexa). The results are summarized in Figure 4. In contrast to 3PGK, Adk binding causes smaller wavelength shifts of 3 fm, due to Adk molecules being almost twice smaller (44 kDa for 3PGK vs 24 kDa for Adk). This smaller size also causes a weaker response when Adk–Alexa complexes are attached to the sensor both at high and low intensities of WGM. Indeed, the dominant mechanism of local heating is related to the absorption of Alexa molecules followed by heating the Adk–Alexa complex. Alexa-790 dye absorbs WGM radiation and transforms its energy into heat via nonradiative relaxation. The quantum yield of luminescence of Alexa in solution is about 10%; however, protein–Alexa complexes bound to the sensor have their luminescence quenched. Therefore, we expect the quantum yield to be of units of percent and hence the energy absorbed from WGM is released as heat. If we consider heat capacity values of 3PGK and Adk are roughly equal and the absorption spectra with efficiency of the Alexa-labeled subject proteins reflecting their molecular weight (see the Supporting Information), then the smaller mass of Adk directly indicates smaller heat absorption; i.e., blue shifts for complexes of 3PGK–Alexa and Adk–Alexa will be observed at different intensities.

Note that the value of ⟨Δλ⟩ for both 3PGK–Alexa and Adk–Alexa complexes is close to −5 fm, also suggesting that Alexa absorption is the ruling mechanism. An important observation is that Adk molecules themselves, without Alexa conjugation, have no absorption around 780 nm and do not demonstrate blue shifts at high intensities. The crucial difference between absorption of 3PGK and Adk at 780 nm is related to the presence of tryptophan in the composition of 3PGK and absence in Adk.

### Tryptamine

**Figure 5:**
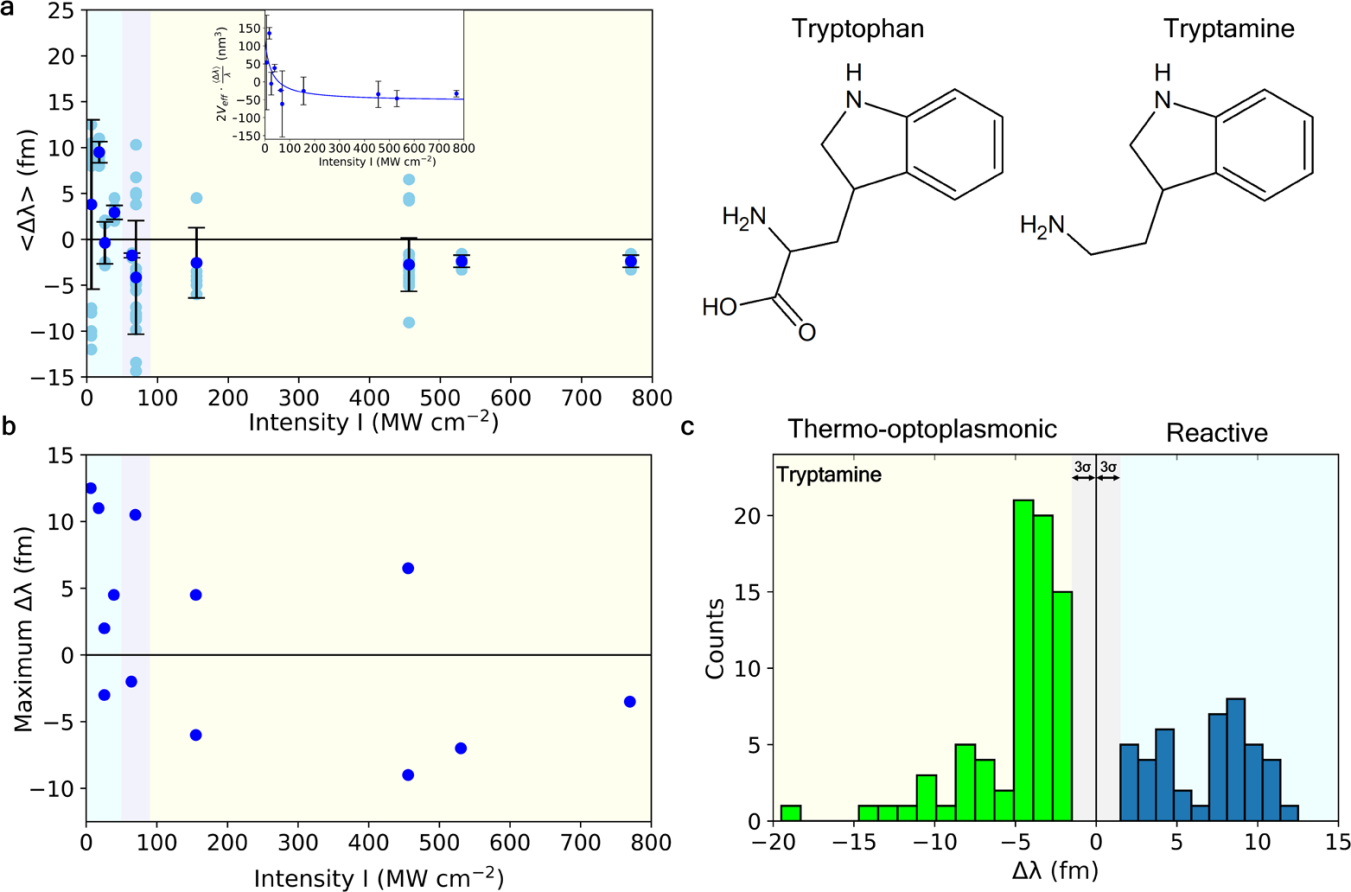
Single-molecule detection of small-molecule tryptamine. (a) Optoplasmonic sensing of single tryptamine molecules (blue circles). Averaged values of WGM wavelength shifts ⟨Δλ⟩ depend on the evanescent intensity *I* of WGM around Au nanorods. Inset: Dependence of 2*V*_eff_·⟨Δλ⟩/λ, with curve fit based on eq 1—*R*^2^ = 0.5613. (b) Maximal wavelength shifts at each intensity *I* of WGM (by absolute values). Maximal wavelength shifts show binding at the tips of nanorods. (c) Histogram of wavelength shifts over 117 individual data points. Both TOP sensing (light blue) and reactive mechanisms (dark blue) are observed, dependent on evanescent intensity. Gray area indicates noise levels of triple the standard deviation, 3σ. Tryptophan and tryptamine structures are presented additionally.

In order to confirm tryptophan’s involvement in TOP sensing, it was appropriate to test the binding of small-molecule tryptophan to the surface of gold nanorods during WGM. However, competition between the amine and carboxyl group (Figure 5) for binding to the nanorods yielded spiked events rather than step-like binding events. To observe Δλ in a step-like manner, a similar small molecule with the same functional group and optical properties was used: tryptamine. At pH 10, the amine group will be deprotonated and a lone pair of electrons will be available for interactions with the nanorod surface.

Upon binding of tryptamine to nanorods, we found a trend of the Δλ profile very similar to that of 3PGK: intensity-dependent changes of the sign of resonant wavelength shifts upon binding. This suggests the TOP sensing effects of 3PGK are likely due to the intrinsic tryptophan residues present in 3PGK, and absence of TOP sensing in Adk due to lack of tryptophan residues. This is likely as a result of effects seen similarly in surface-enhanced resonant Raman scattering (SERRS), where electron transfer from excited plasmons in nanoparticles can form species with visible excitation bands. Sloan-Dennison et al.^35^ demonstrate the ability of tryptophan to undergo these chemical changes when bound to plasmonic nanoparticles during Raman spectroscopy, forming a Trp^–•^ or Trp^+•^ species with absorption bands that span across the visible range, including at 780 nm. Sloan-Dennison et al. favor the formation of the former Trp^–•^ species, describing an electron-capture event when the indole ring of tryptophan is in close proximity to an excited plasmonic nanorod. This previous investigation also demonstrates that electron capture is possible in Trp-containing proteins. We therefore propose the mechanism of apparent forbidden transitions in this case occurs as follows: the ≈780 nm WGM excites plasmons in the nanorods, resulting in electron transfer from the nanorod to the indole ring of tryptophan/tryptamine. This same 780 nm WGM can excite and allow observation of optical transitions in the newly formed Trp^–•^ species, which when relaxing to the ground state releases energy as heat, resulting in the characteristic blue shifts of TOP sensing.

Similarly, the intensity dependence may be due to the requirement to provide sufficient energy to allow electron transfer. This may be most efficient around 70–130 MW cm^–2^ for proteins.^40^ Greater variability of tryptamine than larger protein molecules could also be explained due to its small-molecule nature. This is likely due to tryptamine’s closer position to the nanorod (see Figure S2 in the Supporting Information). Local heating effects and evanescent intensity are more enhanced, resulting in wavelength shifts at the same apparent intensity that can show reactive and TOP sensing.

DFT (density functional theory) calculations, supported by EPR (electron paramagnetic resonance) experiments, show that tryptophan radicals can be intermediates that play important roles in proteins and may even form part of the reaction pathways.^41−43^ With further development of TOP sensing allowing isolation of the shift signals from specific reaction steps at high time resolution, thermo-optoplasmonic sensing may provide a real-time tool for observing transient absorbing states, offering access to sensing transient intermediates of a catalytic or photocatalytic cycle.

### Pure Dye Molecules

**Figure 6:**
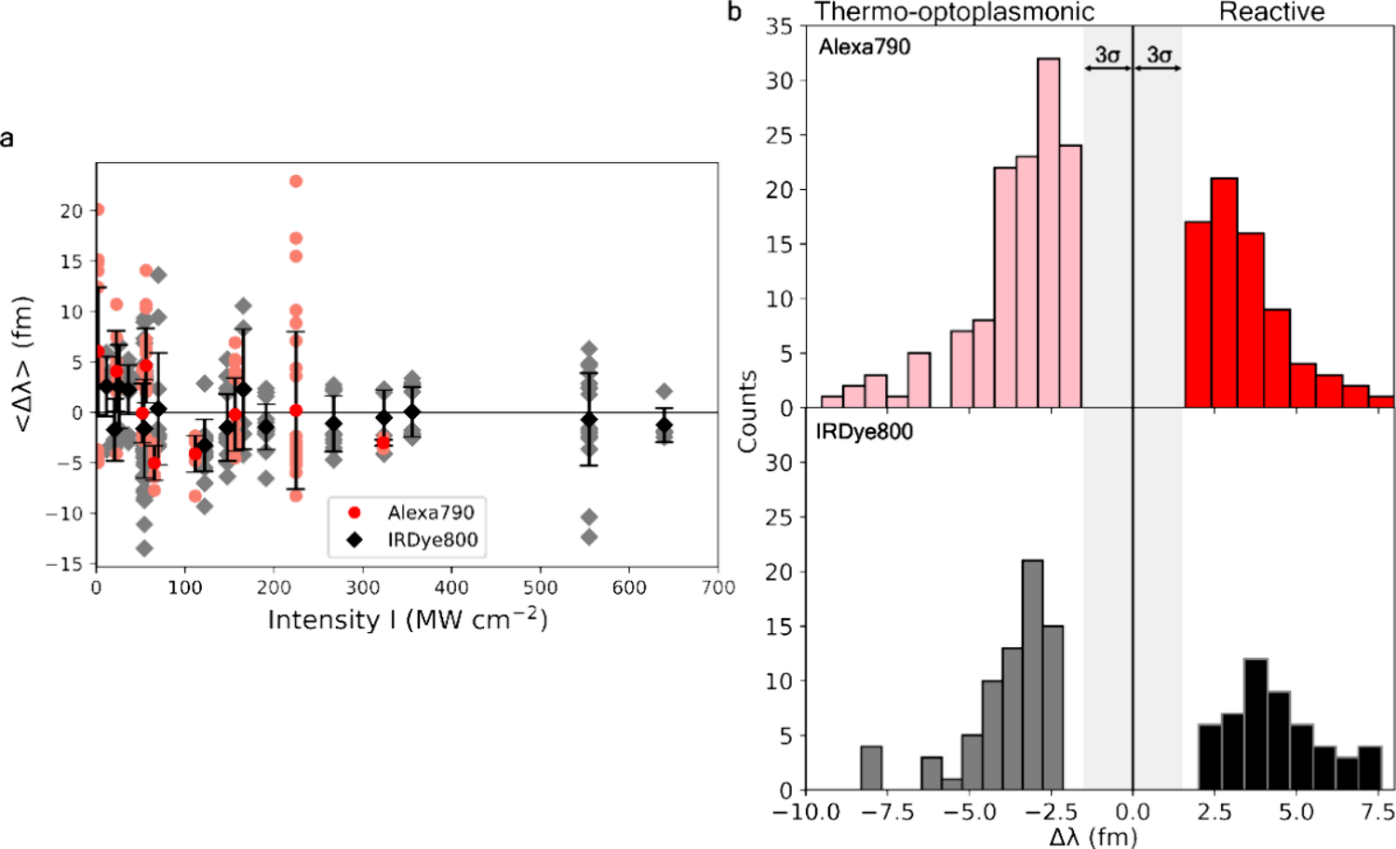
Single-molecule detection of Alexa and IRDye. Resonant wavelength shifts and their averaged values. Alexa molecules (red circles) demonstrate sign-changing behavior: red-shifted resonances at intensities up to 60 MW cm^–2^ and blue-shifts at larger intensities. IRDye molecules (black diamonds) demonstrate blue-shifts even at smaller levels of intensities.

Finally, the TOP sensing mechanism was tested for attachment events of pure Alexa790 and IRDye800 molecules at different intensities. Binding of these molecules to the sensor occurred via interactions of sulfate groups with the gold nanorods (see the Methods). Figure 6 summarizes the results of these control experiments. Upon Alexa molecules binding, sign-changing behavior is observed: red-shifted resonance changes at intensities of up to 60 MW cm^–2^, followed by blue-shifted resonances at larger intensities, i.e., a similar sign-changing trend to 3PGK, 3PGK–Alexa, Adk–Alexa, and tryptamine.

Binding of IRDye molecules demonstrates features of the TOP sensing mechanism also. Resonant wavelength shifts are switched from red to blue at an even smaller level of intensity. The presence of negative shift signals in the dye data sets was enough to confirm our hypothesis. This demonstrates that absorption at the WGM wavelength by molecules attached to the plasmonic nanorods will allow for TOP sensing effects. The larger scattering in these data sets resulting in near-zero averaged values of dye wavelength-shifts at high intensities are likely due to several mechanisms, including the following: (1) Multiple sulfate-mediated binding sites for the nanorod surface with lower affinity, resulting in different transient binding orientations being possible and hence varying nanorod–dipole interactions. (2) IRDye800 and Alexa790 are small molecules able to explore the surface roughness of gold nanorods, similar to tryptamine. (3) Excitations at high intensities resulting in photodecomposition of dye molecules, allowing detection of nonabsorptive regions of the dye, so both reactive and TOP sensing regimes occur.

## Discussion

Thermal effects in WGM nanoparticle and molecular sensing have been previously mentioned in several research works. They mostly use the operational principles of WGM sensors, including tracking mode shifts and changes of their fwhm when sensing particles, micelles, and viruses, and show their different, nontrivial responses. For example, when sensing gold nanoparticles, depending on the wavelength of the WGM, they can demonstrate increasing fwhm and either blue- or red-shifting of resonant wavelengths.^44^ This revealed that it is not just reactive sensing that is possible but also dissipative sensing regimes. The latter can be observed for objects causing losses that may be slightly heated by laser radiation. A similar approach was used in refs (22 and 45) where an object—a polymer molecule or a nanoparticle—placed on a WGM resonator was intentionally heated with a beam, while a second laser was used to monitor WGM resonance changes.

The idea of using whispering-gallery resonators is advancing the powerful technique of photothermal microscopy.^46−52^ The basic principle of this technique is built on registering signals that arise from slight changes of the index of refraction in a sample due to the absorption of a heating light beam. Refractive index changes are measured with a second probing beam, usually of a different color. Even though TOP sensing appears similar to photothermal microscopy and can be associated with the technique described by Goldsmith’s group in 2016,^22^ these two techniques are different. The previously developed technique was used to demonstrate single plasmonic nanoparticle sensing, relying on detecting changes in signals resulting from altering the heating of a nanoparticle by using external lasers to modulate the heating. When coupled with a single (or even several) molecule bound to the nanorod, this yields changes too minute to measure effectively, well within their measurement noise.

Our approach instead focuses on near steady-state heating of the nanorod, where the addition of single molecules can induce significant shifts in the WGM by heating the water at the nanorod’s tip, requiring only a minimal heat flux to effect a noticeable change in the temperature of water and, with that, a detectable refractive index change. As we show, these refractive index changes are detected with very high sensitivity on the optoplasmonic platform, which uses the established method of plasmon-enhanced WGM sensing (reactive sensing) for detecting small polarizability changes on that order.

Contrary to earlier work focusing on WGM microresonators for photothermal spectroscopy, which achieves quantitative determination of absorption cross sections with comparisons to literature values, our experiments attain single-molecule sensitivity. While the previous work accurately extracts absorption cross sections from bulk measurements with absorbing polymers,^45^ our approach leverages the plasmon-enhanced reactive sensing mechanism to achieve a high sensitivity at the level of single molecules, albeit not providing the high accuracy of previous measurements with WGM.

The possibilities of thermo-optoplasmonic sensing can be further developed with appropriate instrumentation, e.g., utilization of different input wavelengths, broadband scanning with different frequencies of whispering-gallery modes, or use of frequency combs.^53^ Initially these tasks seem technically difficult; however, it may be implemented with WGM resonators made with planar architectures or sensor-on-a-chip technologies.^54^ This could be a natural progression of this research and the further development of our experiments. Another direction of use for this mechanism could be in technical improvements of flow measurements in capillaries,^55^ analogous to flow cytometry. This may become an important step in molecular sensing and molecular discrimination by absorption spectra; that can be realized in real-time measurements and with very small amounts of probes. Finally, our technique may be combined with other methods for analysis: recently Yang’s group presented a combination of WGM with Raman spectroscopy.^56^ Such a combined approach allows collection of comprehensive information about analytes per single probe.

As mentioned, the current technique is applicable for a small set of molecules, i.e., molecules with absorption bands close to plasmonic resonances. However, this technique is also limited by the applied intensities. This is mostly related to the potential damage of molecules under test and difficulties in reaching these higher intensities due to optothermal broadening/narrowing effects.^57^

## Conclusions

We have demonstrated single molecule sensing of proteins and proteins labeled with organic dye molecules. It was shown that WGM sensing, improved with plasmonic nanoparticles, is dependent on the intensity of the WGM modes. At low intensity, sensing occurs through the reactive mechanism: red or blue shifts of WGM resonances depending on the polarizability of molecules under test and their refractive index. We established that, at high intensity levels, sensing can occur through a different mechanism: through the absorption of energy by molecules under test followed by heating the surrounding solution, causing blue shifts of the WGM. We have coined this mechanism thermo-optoplasmonic (TOP) sensing. The most exciting part of our results shows that, by using thermo-optoplasmonic sensing, it is possible to define the absorption cross-section of single molecules due to its relation to the WGM wavelength shift.

Equation 1 establishes the relation between the absorption cross-section of molecules and WGM wavelength shifts. This relation serves as a model showing that optoplasmonic sensors can be used as single molecule spectrometers. Further work can be performed via tracking multiple WGM resonances and contribute to studies of photophysical properties of an enormous number of molecules at the single molecule level.

## Methods

### Enzyme Binding to an Optoplasmonic Sensor

The optoplasmonic sensor consists of two major components, the spherical silica microresonator and plasmonic gold nanorods. 6–10 cetrimonium bromide (CTAB) coated gold nanorods (Nanopartz A12-10-780-CTAB) with plasmon resonance at 780 nm were attached to WGM microspheres in 0.02 M HCl, monitored by changes in WGM resonance wavelength (Δλ) and full width at half-maximum (fwhm). Poly-l-lysine-polyeythylene glycol (PLL-PEG) can be used at this stage to prevent nonspecific binding to the surface of the silica microsphere but was found to not be necessary in this study.

3PGK (from *Geobacillus stearothermophilus*; for sequence and purification methods, see the Supporting Information) and 3PGK–Alexa immobilization was performed by modifying the gold-nanorod surface with thiolated nitrilotriacetic acid (NTA). A mixture of 50 μM dithiobis(C2-NTA) (Dojindo D550), 450 μM thiol-dPEG4-acid (Sigma-Aldrich QBD10247), and 250 μM TCEP–HCl (tris(2-carboxyethyl)phosphine–HCl) was incubated for 10 min before mixing in a 1:30 ratio with 50 mM citrate buffer and 1 M NaCl and submerging the microresonator in the solution for a further 20 min in the chamber. The chamber and resonator were washed with 50 mM HEPES. The NTA molecules, on the surface of the nanorods, are then charged with nickel ions by submerging the microresonator in 0.1 M nickel sulfate for 2 min and the chamber finally washed and filled with 50 mM 4-(2-hydroxyethyl)-1-piperazineethanesulfonic acid (HEPES). A 2 μL portion of 0.1 mg mL^–1^ 3PGK or 3PGK–Alexa was then added to the chamber, while monitoring the resonance shift Δλ of WGMs, relying on the Ni-NTA to His-tag interaction for enzyme immobilization onto the nanorod surface.

Adk (from *Aquifex aeolicus*; for the sequence and purification methods, see the Supporting Information) and Adk–Alexa immobilization was performed by direct covalent attachment via gold–thiol interactions, relying on a C-terminal Cys residue. To do so, the chamber was filled with 50 mM TCEP and 50 mM HEPES, and 2 μL of 0.5 mg mL^–1^ Adk or Adk–Alexa was added to the chamber and immobilization monitored by shifts in Δλ of WGMs.

### Tryptamine and Dye Binding to an Optoplasmonic Sensor

Tryptamine binding was performed via amine lone-pair interactions with gold nanorod surfaces at pH 10 in a 50 mM bicarbonate buffer. 2 μL of 1 μM tryptamine (Santa Cruz SC-206065) was added to the chamber in order to observe steps in the Δλ of the WGM.

Alexa Fluor 790 (ThermoFisher A30051) and IRDye 800CW (LI-COR 929-70020) binding to the sensor was performed via interactions of sulfate groups with the gold nanorods. This was performed at pH 7.5 in 50 mM HEPES at concentrations of 13.3–26.7 nM.

### Labeling of 3PGK and Adk with Alexa Fluor 790

3PGK and Adk molecules were labeled with Alexa Fluor 790, using the succinimidyl ester form (ThermoFisher A30051), reacting with free amine groups on the protein surface. Alexa Fluor 790 was dissolved in DMSO to 10 mg mL^–1^ and mixed with a 3 mg/mL solution of protein in 50 mM HEPES and 0.01 M sodium bicarbonate to a final concentration of Alexa Fluor 790 of 0.833 mg/mL. The mixture was incubated while shaking and protected from light for 1 h. To separate protein from free Alexa Fluor 790 and buffer exchanged to an appropriate buffer, size exclusion chromatography (SEC) was performed. SEC was performed using a HiLoad 16/600 Superdex 75pg column (Cytiva 28-9893-33) with an elution buffer of 20 mM HEPES, 150 mM NaCl (pH 7.5) over 1.5 column volumes. Fractions were collected and fractions were selected through correlation of absorption at 280 and 700 nm and confirmed by SDS-PAGE analysis. Selected fractions were pooled and concentrated using Vivaspin 20 3 kDa MWCO PES (Cytiva 28-9323-58) concentrators by centrifugation at 3 kG for 15 min and repeated until the volume was <500 μL.

### Data Processing

A graphical user interface developed in MATLAB for processing the WGM time traces was used similarly to the Supporting Information.^19^ A Labview program was used to record and process the WGM spectra and track the WGM resonance position. Once the WGM time traces were obtained, the data were analyzed for peaks using the MATLAB GUI. First, drift correction caused by slow variations of temperature was applied to remove slow variations of the resonance traces. A first-order Savitzky–Golay filter with a window length depending on the sampling rate was applied to the signal. Second, step-like wavelength traces were analyzed in the MATLAB program to find the resonance shift Δλ values corresponding to proteins binding. Hence, the signal can be close to noise but has a higher amplitude either in wavelength or in fwhm. We quantify useful signals as all steps with an amplitude higher than 3σ (the standard deviation of the background) of that same sample. The value of σ was evaluated by dividing the WGM time trace into windows of *N* points and evaluating the standard deviation of each *N*-point window. Typically, the value of σ is 0.4–0.5 fm, which increases with increased power up to three times. Diagrams (Figures 2 and 4) combined consolidated data of 451 signals from over 50 different experiments. Figure 5 consolidates 117 signals from 10 experiments.

### Evanescent Intensity Calculation

We use a semiempirical approach to evaluate the evanescent intensity *I* of WGM near the nanorods. The intensities used here are limited to *I* < 800 MW cm^–2^ due to optothermal broadening/narrowing during the laser scanning process.^57,58^ The field distribution **E**(**r**) of a specific WGM in microsphere can be numerically computed by using the formulas listed in ref (36). The effective mode volume of WGM is then derived as
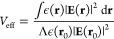
2where ϵ(**r**) denotes the spatial distribution of the relative permittivity and Λ accounts for the local-intensity enhancement factor that arises from the localized surface plasmon resonance (LSPR) of the gold nanorod at the position **r**_0_. The typical value of Λ in this work approximates 800. Greater *I* values could be generated by use of plasmonic nanoparticles with greater near-field enhancement factors and the limiting of optothermal broadening/narrowing effects.^20,58^ It should be noted that the definition of *V*_eff_ here, i.e., eq 2, is different from the one defined based on the maximum light intensity inside the microsphere.^36^ An incident beam with the power *P* pumps the WGM. At the steady state, the intracavity photon number reaches . Here, κ_in_ and κ are the coupling and total loss rates of the microsphere, respectively, *ℏ* is the Planck’s constant, ω = 2*πc*/λ is the angular frequency of the light, and *c* is the speed of light. Thus, the light intensity at the position of the nanorods is given by *I* = *ℏωN*_in_*c*/*V*_eff_.

For each experimental data point shown in the figures, we measured the corresponding microsphere radius *R*, input power *P*, mode wavelength λ, total line width κ, and prism–microsphere coupling efficiency *S*. The effective mode volume *V*_eff_ is numerically computed based on *R*, and the prism–microsphere coupling rate is given by . Then, the evanescent intensity *I* can be evaluated accordingly. As an example, for IRDye800 we measured *R* = 46.5 μm, *P* = 0.19 mW, λ = 780.029083 nm, *S* = 22%, and κ = 503 fm in the experiment. *V*_eff_ and κ_in_ are respectively computed to be *V*_eff_ = 5.9 × 10^–18^ cm^3^ and κ_in_/κ = 0.0584, and then *I* is evaluated to be *I* = 36.2 MW/cm^2^.

### Theoretical Model and Fundamentals for Single Molecule Absorption Spectroscopy

The resonance wavelength shift (from λ to λ′) of WGM induced by the dielectric variation is expressed as^59^
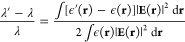
3where ϵ(**r**) corresponds to the relative permittivity in the absence of the perturbation and ϵ′(**r**) denotes the relative permittivity in the presence of the perturbation. Specific to the experiment demonstrated, the resonance shift is caused by (i) the change of the relative permittivity at the location of the ligand protein (approximately, the position of the gold nanorod **r**_0_) and (ii) the change of the relative permittivities of the environment (HEPES or Tris buffer) and microcavity due to the temperature rise (from *T* to *T*′) caused by the protein heating the water and the microsphere. In addition, the LSPR effect has already been included in the relative permittivity ϵ′(**r**). Thus, the resonance shift Δλ = λ′ – λ may be rewritten as (a more detailed derivation can be found in ref (59))
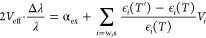
4with the excess polarizability of the protein ,^60^ the relative permittivities of protein ϵ_p_, water ϵ_w_(*T*), and microsphere ϵ_s_(*T*) at the temperature *T*, the protein volume *V*_p_, the effective volume of the heated water *V*_w_, and the effective volume of the heated microsphere *V*_s_. Note: the refractive index of proteins is in the order of *n* = 1.4–1.5, larger than that for water (*n* = 1.33).^15^ This means that α_ex_ for all proteins used in this study (Adk, 3PGK) is always positive when measured in aqueous solution via the reactive sensing regime: the reactive regime response for protein binding will always be positive.

The relative changes of the water and microsphere permittivities caused by the temperature variation Δ*T* = *T*′ – *T* are given by
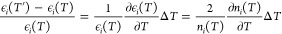
5with the refractive index *n*_*i*_(*T*) of water (*i* = w) or microsphere (*i* = s) at temperature *T*. Equation 4 is then re-expressed as
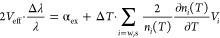
6Since the LSPR only enhances the local field within a small region (hotspot) around the gold nanorod, both effective volumes *V*_w,s_ of the heated water and microsphere are of the order of the hotspot volume . In addition, the change of the refractive index of water with respect to the temperature  K^–1^ is negative and much larger than that of the microsphere  K^–1^, and it gives rise to the negative wavelength shifts.^61^ Thus, the thermo-optic term associated with the microsphere in eq 6 is negligible compared to that of water, and one obtains
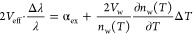
7It is seen that the left side of the above equation is completely related to the microcavity (e.g., the effective mode volume and the resonance shift) and the right side of the above equation includes all environmental perturbations (i.e., the ligand–receptor interactions and the thermal effects). Since *V*_w_ approximates the hotspot volume, we treat it as a water quasi-particle. Due to the negative value of , raising the local water temperature may result in a blue shift of the WGM resonance wavelength.

The heat absorbed by the protein per second is given by *h* = σ_abs_*I* with the absorption cross-section σ_abs_ of the protein and the light intensity *I* at the position of the protein. It should be noted that the LSPR enhancement was taken into account in *I*. Thus, the temperature increment Δ*T* is derived as
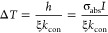
8with the water’s thermal conductivity *k*_con_ and the effective heat transferring length ξ.^25^ Considering the average of the resonance shifts, we arrive at eq 1.

## Supporting Information Available

Survivor functions of 3PGK and tryptamine binding wait time to the optoplasmonic sensor, graphical representation of binding regimes of all molecules, calculation of absorption cross sections of molecules and temperatures of “hot spot” regions, explanation of the effect of nanorod plasmon resonance shift on single-molecule sensing, absorption spectra of molecules, protein sequence and residue components, experimental results without detrending, experimental results at higher intensities, histograms of wavelength shifts per hot spot intensity, and enzyme expression and purification methods (PDF)

## Notes

The preprint of this article was uploaded to www.biorxiv.org. It is available online: Nikita A. Toropov, Matthew C. Houghton, Deshui Yu, Frank Vollmer. Thermo-optoplasmonic single-molecule sensing on optical microcavities. 2023, 2023.12.13.571444. bioRxiv. https://www.biorxiv.org/content/10.1101/2023.12.13.571444v1 (accessed June 7, 2024).
