# Supplementary material for: Thermo-Optoplasmonic Single-Molecule Sensing on Optical Microcavities
Source: ACS Nano. 2024 Jun 26;18(27):17534–46. doi: 10.1021/acsnano.4c00877 (PMC11238588; doi:10.1021/acsnano.4c00877)
Supplement: Supplementary file 1 — nn4c00877_si_001.pdf [file nn4c00877_si_001.pdf]

Supporting Information

Thermo-optoplasmonic single-molecule sensing on optical microcavities

Nikita A. Toropov[1,2,3,†], Matthew C. Houghton[1,4,†], Deshui Yu[5], Frank Vollmer[1,*]

[1]Department of Physics and Astronomy, University of Exeter, Exeter, EX4 4QD, UK

[2]Optoelectronics Research Centre, University of Southampton, Southampton, SO17 1BJ, UK

[3]Qingdao Innovation and Development Center, Harbin Engineering University, Qingdao, Shandong, 266000, China

[4]Department of Life Sciences, University of Bath, Bath, BA2 7AX, UK

[5]National Time Service Center, Chinese Academy of Sciences, Xi'an, 710600, China

Confirmation of Single-Molecule Binding Regime

When collecting all binding shifts where $\Delta\lambda > 3\sigma$, we find the wait time (Δt) to fit a single-exponential following $P(0, \Delta t) = e^{-R\Delta t}$ where R is the rate constant of binding, as also outlined in other publications **(1)**. These survivor functions (Fig. S1) demonstrate binding to be on the single-molecule level. Deviations from a perfect exponential decay may be due to non-homogenous distribution of analyte in the solution due to temperature or diffusion variations.

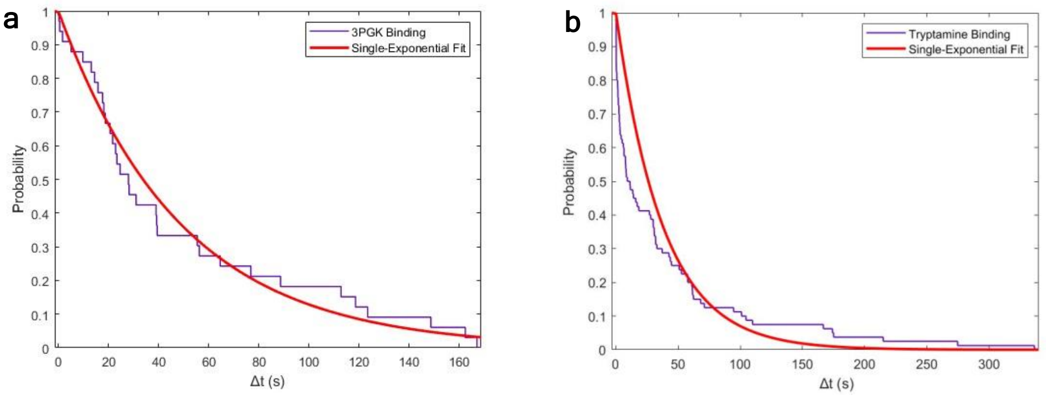

Fig. S1 | a. Survivor function of 3PGK binding wait time (Δt) to the optoplasmonic sensor (purple) showing exponential decay where $P(0, \Delta t) = e^{-R\Delta t}$ (red). **b.** Survivor function of tryptamine binding wait time to the optoplasmonic sensor (purple) showing exponential decay (red).

The molecules tested here bind by slightly different methods, dependent on their specific chemistries. 3PGK proteins bind via a His-tag-Ni-NTA linkage, Adk via direct cysteine sulphur-gold bonding, tryptamine via amine-gold interactions and IRDye800Cw and Alexa790 via sulfate-gold interactions (Fig. S2). These binding regimes are specific and non-random, ensuring they bind in a single orientation.

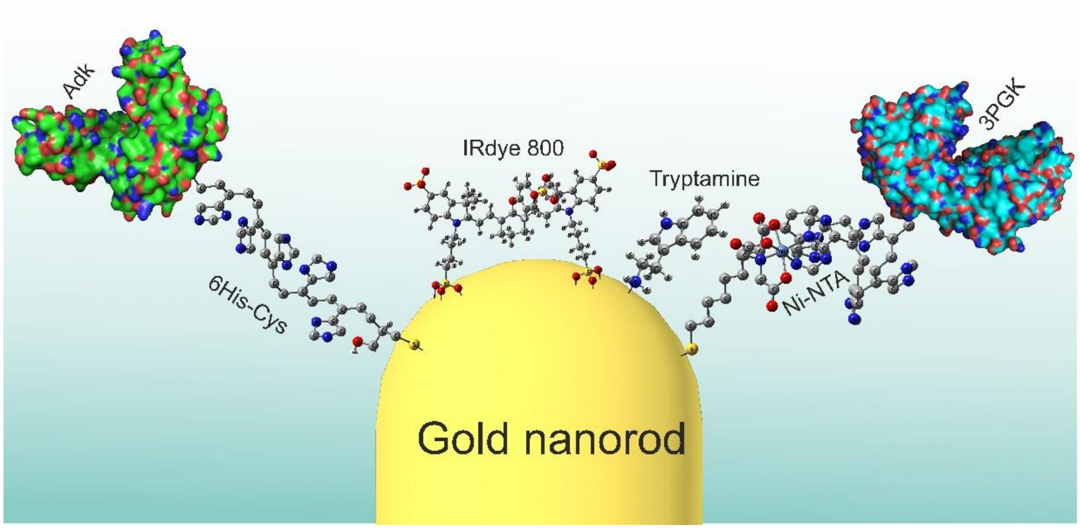

Fig. S2 | Binding regime of all molecules tested. Alexa790 has no published structure so cannot be presented here, but linkages are proposed to be similar to those of IRDye800Cw.

Absorption Cross-Section Calculation

The absorption cross-section σ_{abs} was directly estimated with the following formula:

$$\sigma_{abs} = \frac{2.3 \cdot 10^3}{N_A} \varepsilon,$$

where ε is the molar extinction coefficient.

The degree of labelling was calculated using equations supplied by ThermoFisher **(2)**, calculated as 0.65 and 0.76 for 3PGK-Alexa and Adk-Alexa, respectively.

$$A_{280nm}^{Alexa790} = A_{785} * CF_{280nm} \qquad CF_{280:785nm}^{Alexa790} = 0.08$$

$$A_{280nm}^{Protein} = A_{280nm} - A_{280nm}^{Alexa790}$$

$$[Protein] = \frac{A_{280nm}^{Protein}}{\varepsilon_{280nm}^{Protein} * L}$$

$$DoL = \frac{A_{785nm}}{[Protein] * L}$$

The extinction coefficients of Alexa790 and protein-Alexa790 conjugates were calculated by the below equation:

$$[Alexa790] = [Protein] * DoL$$

$$\frac{A_{280nm}^{Alexa790}}{[Alexa790]*L} = \varepsilon_{280nm}^{Alexa790}$$

$$\varepsilon_{280nm}^{Protein-Alexa790} = \varepsilon_{280nm}^{Alexa790} + \varepsilon_{280nm}^{Protein}$$

Where units of the values are:

Absorption (A): AU; Conversion Factor ($CF_{280:785nm}$): AU; Concentration ([x]): M; Pathlength (L): cm; Degree of Labelling (DoL): AU; Extinction coefficient (ε): $cm^{-1}M^{-1}$.

Temperature of the 'Hot Spot' Region

We assessed the changes of temperature of nanorods when heated by radiation. Indeed, using the thermal diffusion equation, the surface temperature of a nanoparticle may be estimated with its absorption cross-section and size, incident power and thermal conductivity of the surrounding medium **(3,4).** For gold nanoparticles used in an aqueous medium at WGM intensities of values we work with and typical values of absorption cross-sections of gold nanorods of ~10^{-18} m^2 **(**taken from **5,6)**, their temperature is changed within 20 K (Fig. S3).

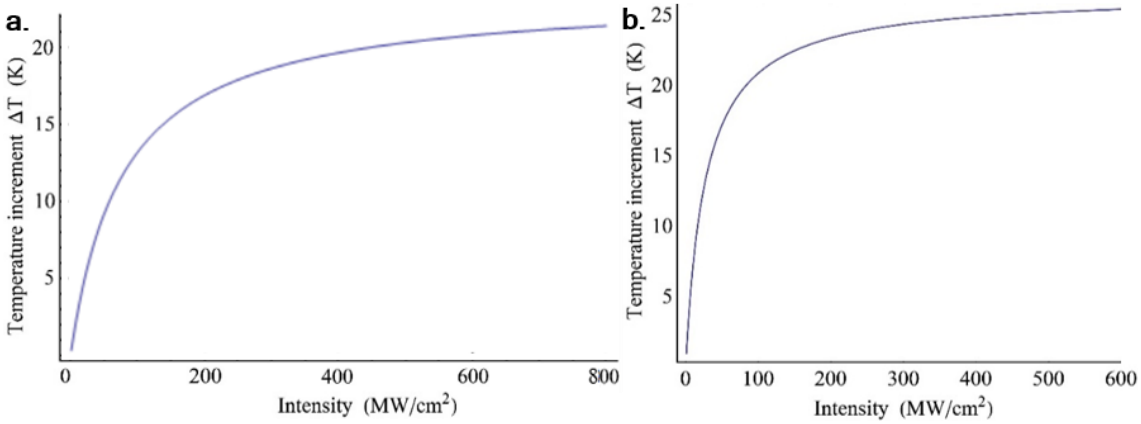

Fig. S3 | a. Temperature of the locally heated area where molecules attach vs electric field intensity in this area of **a.** the plasmonic nanorod and **b.** Alexa790.

Effect of nanorod plasmon resonance shift on single-molecule sensing

The localized surface plasmon resonance (LSPR) of gold nanorods strongly enhances the near-field light intensity (at the hot spot) by a factor of Λ. In the main text, the local electric-field intensity I has already included the enhancement factor Λ. That is, in the absence of gold nanorods, the local electric-field intensity is given by (I/Λ). For the typical size of gold nanorods with a length of 38 nm and a diameter of 10 nm in this work, we have $\Lambda = 800$ and the absorption cross-section of gold nanorod is derived as $\sigma_{\text{abs}}^{\text{Gold Nanorod}} = 2.7 \times 10^{-13}$ cm^2 at 780 nm. Indeed, such a large absorption cross-section directly results from the LSPR. Thus, the energy absorbed by a gold nanorod takes the form $E_{\text{Gold Nanorod}} = \sigma_{\text{abs}}^{\text{Gold Nanorod}} I/\Lambda$. Since $\sigma_{\text{abs}}^{\text{Gold Nanorod}}$ has already taken into account the LSPR (i.e., the enhancement factor Λ), the electric field (light) acting on the gold nanorod should be (I/Λ), rather than I. In comparison, the light energy absorbed by a molecule (for example, 3PGK) is given by $E_{\text{3PGK}} = \sigma_{\text{abs}}^{\text{3PGK}} I$ with the absorption cross-section of 3PGK $\sigma_{\text{abs}}^{\text{3PGK}} = 7.5 \times 10^{-16}$ cm^2 at 780 nm (see the main text). We have $E_{\text{3PGK}}/E_{\text{Gold Nanorod}} = \Lambda\sigma_{\text{abs}}^{\text{3PGK}}/\sigma_{\text{abs}}^{\text{Gold Nanorod}} = 2.2$. Thus, the energy is mainly absorbed by the molecule, rather than the gold nanorod.

In addition, the change of the absorption cross-section of the gold nanorod caused by a plasmon resonance shift of 1 nm is computed to be $\Delta\sigma_{\text{abs}}^{\text{Gold Nanorod}} = 0.04 \times 10^{-13}$ cm^2. Although $\Delta\sigma_{\text{abs}}^{\text{Gold Nanorod}}$ is six times of $\sigma_{\text{abs}}^{\text{3PGK}}$, i.e., $\Delta\sigma_{\text{abs}}^{\text{Gold Nanorod}}/\sigma_{\text{abs}}^{\text{3PGK}} = 6$, the change of the energy absorbed by the gold nanorod $\Delta E_{\text{Gold Nanorod}} = \Delta\sigma_{\text{abs}}^{\text{Gold Nanorod}} I/\Lambda$ is negligible compared to E_{3PGK}, i.e., $E_{\text{3PGK}}/\Delta E_{\text{Gold Nanorod}} = 136$. Therefore, the effect of the change of the light energy absorbed and ultimately dissipated by gold nanorods on the single-molecule-induced resonance shift can be safely neglected.

Absorption spectra of subject proteins

The absorption of 3PGK, 3PGK–Alexa, Adk, Adk–Alexa and tryptamine were evaluated by absorption spectroscopy using a Horiba Duetta absorption spectrometer (Fig. S4).

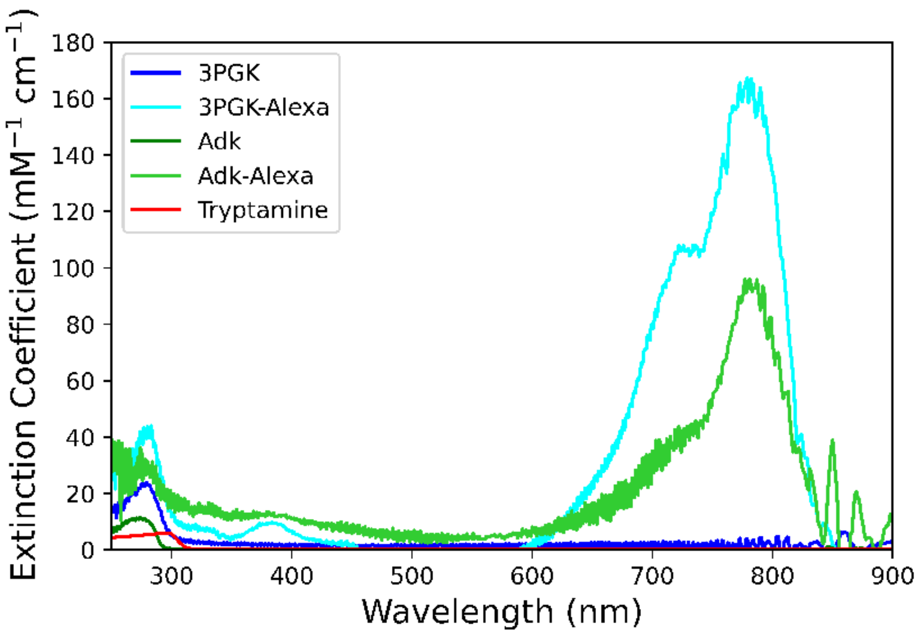

Fig. S4 | Absorption spectra of the molecules under test.

Protein Sequence and Residue Components

Adenylate Kinase (Adk) from *Aquifex aeolicus*:

MILVFLGPPGAGKGTQAKRLAKEKGFVHISTGDILREAVQKGTPLGKKAKEYMERGELV
PDDLIIALIEEVFPKHGNVIFDGFPRTVKQAEALDEMLEKKGLKVDHVLLFEVPDEVVIER
LSGRRINPETGEVYHVKYNPPPPGVKVIQREDDKPEVIKKRLEVYREQTAPLIEYYKKKGI
LRIIDASKPVEEVYRQVLEVIGDGSHHHHHHC*

3-Phosphoglycerate Kinase (3PGK) from *Geobacillus stearothermophilus:*

MHHHHHHNKKTIRDVDVRGKRVFCRVDFNVPMEQGAITDDTRIRAALPTIRYLIEHGAK
VILASHLGRPKGKVVEELRLDAVAKRLGELLERPVAKTNEAVGDEVKAAVDRLNEGDV
LLLENVRFYPGEEKNDPELAKAFAELADLYVNDAFGAAHRAHASTEGIAHYLPAVAGFL
MEKELEVLGKALSNPDRPFTAIIGGAKVKDKIGVIDNLLEKVDNLIIGGGLAYTFVKALG
HDVGKSLLEEDKIELAKSFMEKAKEKGVRFYMPVDVVVADRFANDANTKVVPIDAIPA
DWSALDIGPKTRELYRDVIRESKLVVWNGPMGVFEMDAFAHGTKAIAEALAEALDTYS
VIGGGDSAAAVEKFGLADKMDHISTGGGASLEFMEGKQLPGVVALEDKHHHHHH

Table S1 | Residues present in designed Adk and 3PGK proteins.

**Adk**	**3PGK**
Ala (A) 10	Ala (A) 49
Arg (R) 12	Arg (R) 20
Asn (N) 3	Asn (N) 13
Asp (D) 11	Asp (D) 31
Cys (C) 1	Cys (C) 1
Gln (Q) 6	Gln (Q) 2
Glu (E) 23	Glu (E) 33
Gly (G) 18	Gly (G) 35
His (H) 10	His (H) 20
Ile (I) 16	Ile (I) 21
Leu (L) 18	Leu (L) 38
Lys (K) 22	Lys (K) 32
Met (M) 2	Met (M) 9
Phe (F) 6	Phe (F) 15
Pro (P) 15	Pro (P) 15
Ser (S) 4	Ser (S) 11
Thr (T) 6	Thr (T) 13
**Trp (W) 0**	**Trp (W) 2**
Tyr (Y) 7	Tyr (Y) 8
Val (V) 22	Val (V) 38
Pyl (O) 0	Pyl (O) 0
Sec (U) 0	Sec (U) 0

Testing 3PGK molecules at higher intensities

TOP sensing is limited by the intensity of WGM used for probing. At the current stage, it seems unnecessary to define maximum intensities allowed for experiments since they appear to be different for each molecule and does not provide additional information about absorption. Nevertheless, we performed experiments at higher intensities (I = 625 MW cm^{-2}) that demonstrate anomalous large shifts (av. = 41.0 +/- 51.3) which are related to a different undescribed process: most likely protein aggregation (Fig. S5).

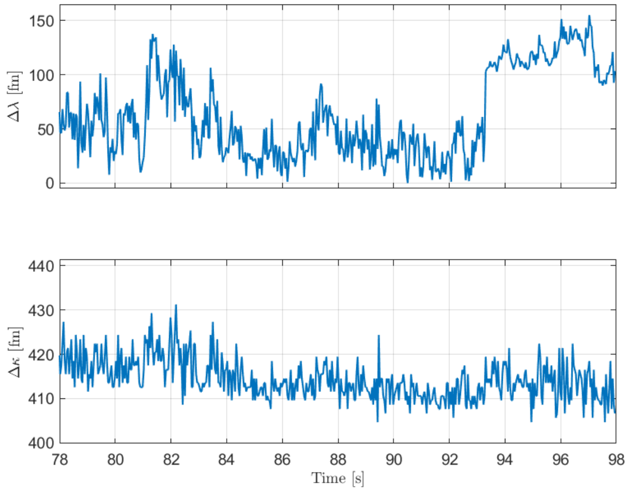

Fig. S5 | Large wavelength shifts (Δλ) at I = 625 MW cm^{-2}. FWHM (Δκ) is also provided.

Enzyme Binding (Detrended)

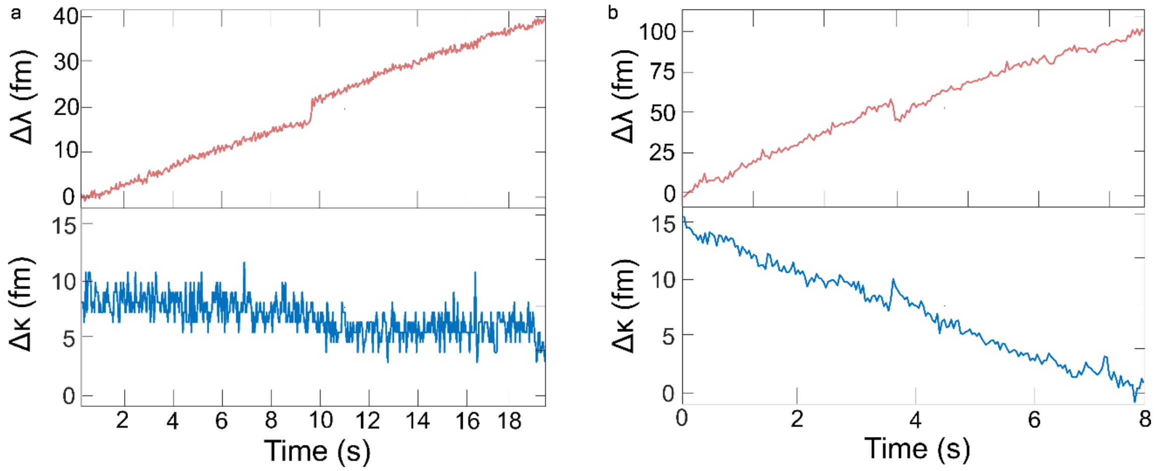

Fig. S6 | Raw data of **a.** 3PGK binding in the reactive sensing regime, showing a positive wavelength change, and **b.** 3PGK binding in the TOP sensing regime, showing a negative wavelength change and positive FWHM change. No detrending was performed on the data for these figures. Wavelength and FWHM shifts were extracted from non-detrended data such as this.

Wavelength Shifts per Hotspot Intensity

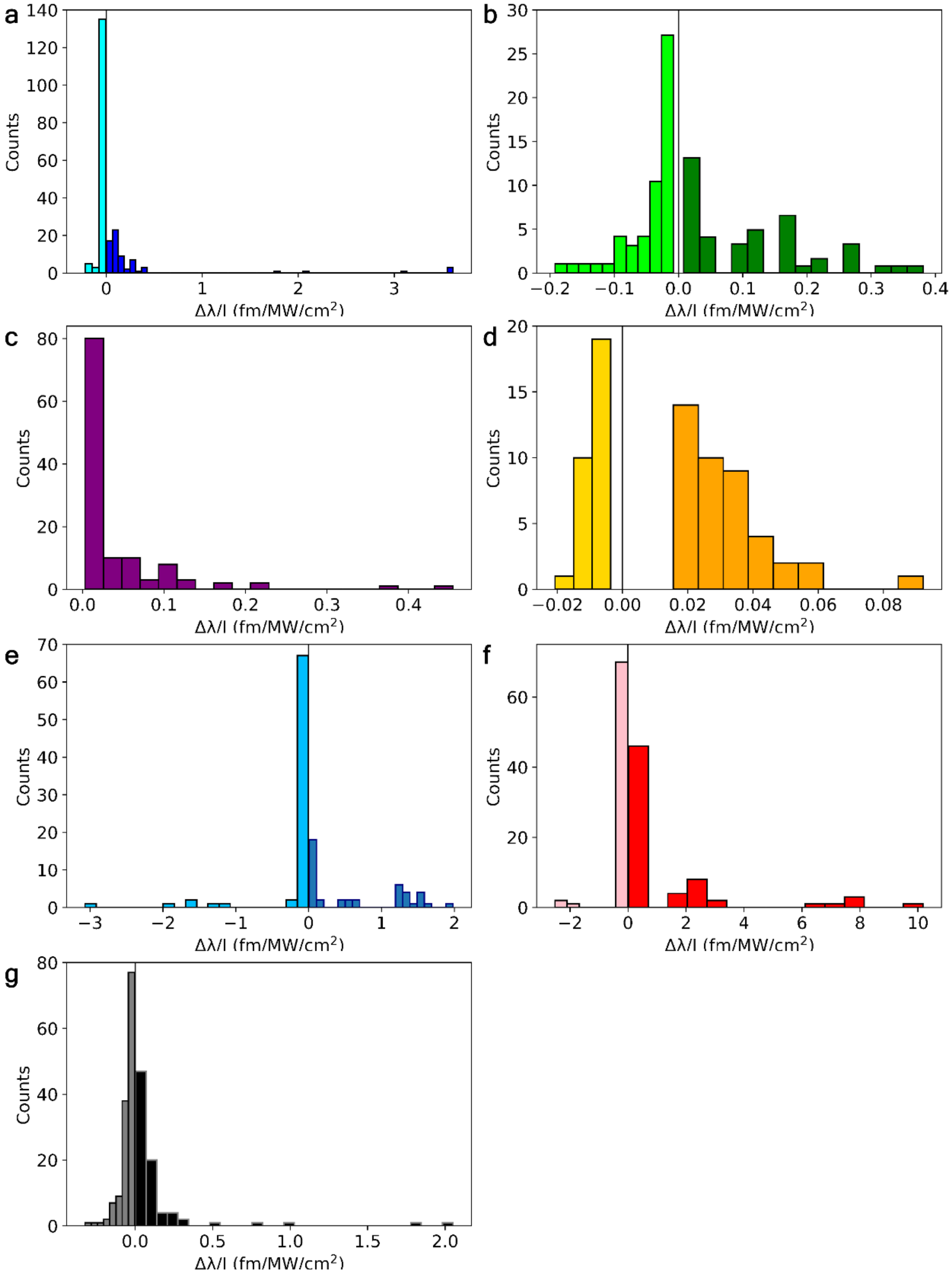

Fig. S7 | Histograms of wavelength shifts per hotspot intensity ($\Delta\lambda/I$) of **a.** 3PGK, **b.** 3PGK-Alexa, **c.** Adk, **d.** Adk-Alexa, **e.** Tryptamine, **f.** Alexa790, and **g.** IRDye800.

Enzyme Expression and Purification Methods

3PGK and Adk fragments, DNA sequences optimised for E. coli expression, were prepared in pET30a(+) plasmids by GenScript. Both were separately transformed into BL21(DE3) E. coli (NEB) by heat-shock and grown on agar at 37 °C overnight, selected for with 50 µg/mL kanamycin. Singular colonies were transferred to an LB media containing 50 µg/mL kanamycin and grown at 37 °C until an OD800 of 0.4-0.8. 400 µM IPTG was used to induce protein expression at 20 °C for 16 hrs.

Cells were harvested by centrifugation at 4 kG for 30 mins at 4 °C and cell pellet resuspended in 5 mL of HisA buffer (50 mM Tris-HCl, 300 mM NaCl, 20 mM Imidazole, pH 7.5). The cell suspension was sonicated on a pulse setting at 60 % amplitude for 10 s with a 10 s rest over 5-10 mins. The lysate was centrifuged at 26 kG for 30 mins at 4 °C and soluble fraction retained for purification.

Purification of the soluble fraction was performed in two steps: an initial purification by Nickel-immobilised metal affinity chromatography (Ni-IMAC), followed by gel filtration. Both were performed on an ÄKTA pure™ 150 (Cytiva) at 4 °C. The soluble fraction from expression was passed through a 0.22 µm Millex®-GS sterile filter and injected into a 5 mL sample loop. A HisTrapFF-1ml column (Cytiva GE17-5319-01) was used with a binding buffer of HisA and elution buffer of His B (50 mM Tris-HCl, 300 mM NaCl, 500 mM Imidazole, pH 7.5). Elution was performed with a concentration gradient of increasing HisB:HisA ratio from 0-100% over 10 mins at 1 mL/min, collecting 2 mL aliquots. Fractions containing the desired protein were selected by SDS-PAGE analysis and pooled for gel filtration before concentrating to 5 mL using a Vivaspin 20 3,000 MWCO polyethersulfone centrifugal concentrator (Sartorious VS2092) at 3 kG and 4 °C.

Gel filtration was performed using a Superdex 75 16/600 SEC column (Cytiva GE28-9893-33) and running buffer of 20 mM Tris-HCl, 150 mM NaCl, pH 7.5 at 0.5 mL/min, collecting 2 mL fractions. Fractions with protein were selected via SDS-PAGE and pooled, before concentrating again with centrifugal concentrators (as above) to a desired concentration.

References

(1) Baaske, M. D. & Vollmer, F. Optical observation of single atomic ions interacting with plasmonic nanorods in aqueous solution. Nature Photonics 10, 733-739 (2016).

(2) Amine-Reactive Probes (ThermoFisher Scientific). https://www.thermofisher.com/document-connect/document-connect.html?url=https://assets.thermofisher.com/TFS-Assets%2FLSG%2Fmanuals%2Fmp00143.pdf (Accessed 2024-03-26).

(3) Andronaki, S. A. & Vartanyan, T. A. Numerical simulation of steady-state optical heating of aluminum nanoparticles, in 2018 International Conference Laser Optics (ICLO). P. 417-417.

(4) Baffou, G., Quidant, R. & García de Abajo, F. J. Nanoscale Control of Optical Heating in Complex Plasmonic Systems. ACS Nano 4, 709-716 (2010). https://doi.org:10.1021/nn901144d

(5) Jain, P. K., Lee, K. S., El-Sayed, I. H. & El-Sayed, M. A. Calculated Absorption and Scattering Properties of Gold Nanoparticles of Different Size, Shape, and Composition: Applications in Biological Imaging and Biomedicine. The Journal of Physical Chemistry B 110, 7238-7248 (2006). https://doi.org:10.1021/jp057170o

(6) Alrahili, M., Savchuk, V., McNear, K. & Pinchuk, A. Absorption cross section of gold nanoparticles based on NIR laser heating and thermodynamic calculations. Scientific Reports 10, 18790 (2020). https://doi.org:10.1038/s41598-020-75895-9
